# Concurrence of symmetrical peripheral gangrene and venous limb gangrene following polytrauma: a case report

**DOI:** 10.1186/s13256-018-1684-1

**Published:** 2018-05-19

**Authors:** Jih Huei Tan, Yuzaidi Mohamad, Chor Lip Henry Tan, Mahazir Kassim, Theodore E. Warkentin

**Affiliations:** 1 Department of General Surgery, Pusat Perubatan Universiti Kebangsaan Malaysia, Cheras, Malaysia; 2 Department of General Surgery, Hospital Sultanah Aminah, Johor Bahru, Malaysia; 3 Department of Anaesthesiology and Intensive Care, Hospital Sultanah Aminah, Johor Bahru, Malaysia; 4 Department of Pathology and Molecular Medicine, McMaster University, Hamilton, ON Canada; 5 Hamilton Regional Laboratory Medicine Program, Hamilton General Hospital, Room 1-270B, 237 Barton St. East, Hamilton, ON L8L 2X2 Canada

**Keywords:** Amputations, Deep vein thrombosis, Symmetrical peripheral gangrene, Venous limb gangrene

## Abstract

**Background:**

Symmetrical peripheral gangrene is characterized as acral (distal extremity) ischemic limb injury affecting two or more extremities, without large vessel obstruction, typically in a symmetrical fashion. Risk factors include hypotension, disseminated intravascular coagulation, and acute ischemic hepatitis (“shock liver”). In contrast, venous limb gangrene is characterized by acral ischemic injury occurring in a limb with deep vein thrombosis. Both symmetrical peripheral gangrene and venous limb gangrene present as acral limb ischemic necrosis despite presence of arterial pulses. The coexistence of symmetrical peripheral gangrene and venous limb gangrene is rare, with potential to provide pathophysiological insights.

**Case presentation:**

A 42-year-old Chinese man presented with polytrauma (severe head injury, lung contusions, and right femur fracture). Emergency craniotomy and debridement of right thigh wound were performed on presentation. Intraoperative hypotension secondary to bleeding was complicated by transient need for vasopressors and acute liver enzyme elevation indicating shock liver. Beginning on postoperative day 5, he developed an acute platelet count fall (from 559 to 250 × 10^9^/L over 3 days) associated with left iliofemoral deep vein thrombosis that evolved to bilateral lower limb ischemic necrosis; ultimately, the extent of limb ischemic injury was greater in the left (requiring below-knee amputation) versus the right (transmetatarsal amputation). As the presence of deep vein thrombosis is a key feature known to localize microthrombosis and hence ischemic injury in venous limb gangrene, the concurrence of unilateral lower limb deep vein thrombosis in a typical clinical setting of symmetrical peripheral gangrene (hypotension, proximate shock liver, platelet count fall consistent with disseminated intravascular coagulation) helps to explain asymmetric limb injury – manifesting as a greater degree of ischemic necrosis and extent of amputation in the limb affected by deep vein thrombosis – in a patient whose clinical picture otherwise resembled symmetrical peripheral gangrene.

**Conclusions:**

Concurrence of unilateral lower limb deep vein thrombosis in a typical clinical setting of symmetrical peripheral gangrene is a potential explanation for greater extent of acral ischemic injury in the limb affected by deep vein thrombosis.

## Background

Symmetrical peripheral gangrene (SPG) is defined as distal ischemic damage in two or more extremities, without large vessel obstruction or vasculitis, typically in a largely symmetrical fashion [1, 2]. Disseminated intravascular coagulation (DIC), usually occurring in the setting of hypotension and shock (septic, cardiogenic, hypovolemic), is the major contributing factor [1–3]. Recently, a role for preceding acute ischemic hepatitis (“shock liver”) has been identified in approximately 90% of patients with SPG; the relevant concept is that acute liver injury predisposes to microthrombosis, and resulting ischemic limb injury, through decreased hepatic production of the key natural anticoagulants, protein C and antithrombin [1, 4]. SPG is associated with a mortality rate of up to 40% [5].

In comparison with SPG, venous limb gangrene (VLG) results from a different pathogenesis, as by definition it involves unilateral deep vein thrombosis (DVT) in the limb affected by acral (distal extremity) ischemic necrosis. Patients typically have associated DIC, and the concept is that large vein thrombosis predisposes to microthrombosis in the same limb due to decreased blood flow and/or direct contiguous extension of thrombosis [1]. Since DVT most often occurs in one limb, VLG usually presents with unilateral limb ischemic necrosis, whereas by definition SPG usually affects two (or four) limbs in a mainly symmetrical fashion [1]. However, common to both VLG and SPG, patients present with the typical clinical picture of “ischemic limb gangrene with pulses” [1].

Usually, SPG occurs without associated DVT [1]. However, we present a case of critical illness complicated by hypotension, shock liver, and thrombocytopenia, where the patient developed SPG together with acute left iliofemoral DVT. The patient exhibited a disproportionate degree of injury, as shown by the higher level of amputation required in the limb that was also affected by DVT. The clinical picture indicates concurrence of SPG and VLG and suggests that the presence of DVT can modulate the clinical picture of SPG. To the best of our knowledge, a similar case has not been reported in the literature.

## Case presentation

A 42-year-old Chinese man riding a motorbike sustained blunt trauma following collision with a lorry. He had decreased level of consciousness and a nosebleed when found lying at the roadside. On arrival at the emergency department, his Glasgow Coma Scale (GCS) was E2V2M4, right pupil was 4 mm and left pupil was 2 mm with sluggish reactions. His blood pressure (BP) was 169/126 mmHg, heart rate 89 beats/minute, and arterial oxygen saturation (SaO_2_) was 80% (room air). He was intubated and resuscitated following principles of Advanced Trauma Life Support. A computed tomography (CT) scan of his brain showed right frontal intraparenchymal bleeding (3.5 × 1.5 × 2.6 cm), right cerebral sulci effacement, 0.9 cm midline shift, multiple extradural hemorrhages of 1 cm diameter, and multiple small contusions in bilateral frontal and right temporal regions. A thoracic and abdominal contrast CT scan showed bilateral lung contusions with multiple rib fractures. Pelvic and right femur X-rays showed fractures of the right neck of femur and midshaft with open wound (Gustilo grade two). Bilateral pedal pulses were palpable.

Within 3 hours post-trauma, emergency decompressive craniectomy and right femur wound debridement were performed. Intraoperatively, his BP dropped to 80/50 mmHg due to bleeding from his right femur and craniectomy, and noradrenaline infusion was started (highest dose, 0.16 mcg/kg per minute). Postoperatively, his alanine aminotransferase level measured 182 U/mL (reference range, 10–40) and aspartate aminotransferase level measured 405 U/mL (reference range, 10–40), consistent with hypotension-related shock liver in this clinical context.

Estimated intraoperative total blood loss was 1 l. Two units of packed cells were transfused. Postoperatively, he was ventilated in an intensive care unit, and intravenously administered fentanyl and propofol were maintained for 24 hours. Following stoppage of sedation, noradrenaline was weaned off (on postoperative day 2). However, his subsequent GCS recovery was poor (E1VtM4). Unfractionated heparin (5000 IU twice daily by subcutaneous injection) was commenced on the third day for DVT prophylaxis, as risk of bleeding from injured organs was deemed to be low at that time.

On day 5, he developed bilateral lower limb swelling extending into both thighs, more marked on his left thigh. Bluish discoloration of all toes was noted with presence of bilateral pedal Doppler signals. His fingers were normal. Duplex scanning of the deep veins of his lower limbs showed left external iliac and femoral vein thrombosis, with no DVT on the right side. CT angiography revealed good opacification of arteries to the small vessels distal to both ankles, consistent with bilateral distal microvascular thrombosis; that is, the radiological picture was consistent with SPG. Low-dose heparin thromboprophylaxis was replaced by therapeutic-dose intravenously administered heparin upon diagnosis of DVT of his left lower limb; at the same time, hemodialysis was commenced.

**Fig. 1:**
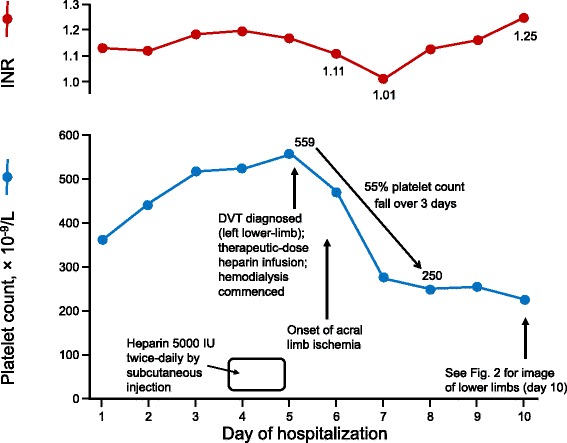
Changes in platelet count and international normalized ratio from hospital day 1 to day 10. The beginning of the 55% platelet count decline coincided with the onset of progressive acral ischemia. *DVT* deep vein thrombosis, *INR* international normalized ratio

His platelet count fell abruptly from 559 × 10^9^/L on the fifth hospital day (measured shortly before onset of limb ischemia) to 250 on the eighth day, a decline of approximately 55% (Fig. 1). Progressive acral limb ischemia occurred during this time period. The platelet count fall (starting on the third day of heparin therapy) was deemed too soon to indicate immune heparin-induced thrombocytopenia (HIT) on clinical grounds (although testing for HIT antibodies was not locally available).

At the time of onset of limb ischemia (day 6 of hospitalization), blood testing showed: hemoglobin, 75 g/L; white cell count, 12.7 × 10^9^/L; C-reactive protein (CRP), 169.7 mg/l (reference range, < 10); erythrocyte sedimentation rate, 61 mm (reference range, 0–22); prothrombin time, 11.6 (reference range, 11.5–15.5 seconds); international normalized ratio (INR), 1.11 (reference range, < 1.4); and activated partial thromboplastin time, 43.3 seconds (reference range, 30–40 seconds). He had acute renal failure with urea of 43.8 mmol/L (reference range, 2.8–8.1) and serum creatinine of 357 μmol/L (reference range, 62–106), which resolved after a week. Blood cultures were all negative.

**Fig. 2:**
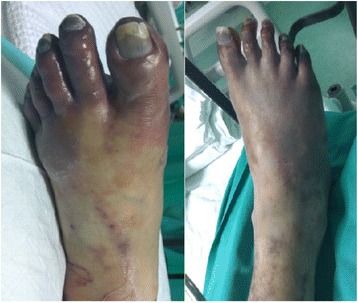
Right foot and left foot of this patient, showing that all his toes are gangrenous (photo taken at day 10)

Thrombectomy was done by the vascular surgeon via femoral vein. Two days post-thrombectomy, left iliofemoral venous thrombosis recurred, and repeat thrombectomy was performed. Despite heparin treatment and repeat thrombectomies, toe ischemia progressed to more extensive lower limb ischemic gangrene (Fig. 2 showing clinical appearance on day 10). Right transmetatarsal and left below-knee amputations were performed. However, he died during his sixth week of hospitalization due to pneumonia and infected bed sore.

## Discussion and conclusions

SPG is a clinical syndrome with largely symmetrical acral (distal extremity) necrosis despite palpable or Doppler-identifiable arterial pulses, more often involving the lower limbs; the upper limbs are additionally affected in only one-third of cases [1]. VLG is defined as acral necrosis of an extremity in the setting of an acute DVT affecting the same limb [1]. Both entities share a similar pathophysiological feature in that there is microthrombosis associated with a DIC state and natural anticoagulant failure [1, 6].

SPG has been reported in a variety of clinical scenarios. Its common associations are septic and cardiogenic shock [1]. Other reported clinical settings of SPG include malaria [7], dengue fever [8], post-cardiac surgery [9], pancreatitis [10], post-partum [11], and following snake bite [12]. All of these conditions are often associated with a proinflammatory state; the inflammatory cytokines trigger DIC and potential for microthrombosis. Low flow state secondary to hypotension or cardiac failure together with acquired protein C and antithrombin depletion further perpetuate acral microthrombosis [1]. Our polytrauma patient had several risk factors for SPG, including multiple injuries inducing a proinflammatory response (high CRP level) and acute hepatic dysfunction (shock liver) as a contributing factor in subsequent natural anticoagulant depletion (although we were not able to measure these natural anticoagulant factors in our hospital in Malaysia). Further depletion of protein C has been reported to occur in the setting of polytrauma [13].

The concurrent presence of DVT in one affected limb adds additional complexity to the case, as DVT complicating SPG is rare. Although both entities can be associated with DIC, the underlying triggers of DIC tend to differ. Patients with VLG usually present with unilateral proximal DVT in association with an underlying hypercoagulability state [1], such as HIT [14, 15] or cancer-related consumptive coagulopathy [16], and often with protein C depletion induced by warfarin [14, 16]. A case of VLG after initiation of rivaroxaban has also been reported [17].

In comparison with previously reported cases of SPG, the presence of DVT with VLG in our current case is unusual. He did not have an underlying malignancy, warfarin intake, or elevated INR. However, our patient did develop an abrupt platelet count fall from 559 to 250 that coincided with the development of ischemic limb injury. Although we cannot definitively rule out the possibility of HIT (as testing for HIT antibodies could not be performed), the early timing of onset of thrombocytopenia in relation to start of heparin prophylaxis makes this drug reaction unlikely [18]. However, the abrupt onset of platelet count fall, presumably reflecting a degree of platelet activation, irrespective of cause, probably played an important role in predisposing our patient to microvascular thrombosis.

There is no single effective therapy to treat both SPG and VLG. Treatment for SPG includes avoidance/dose-reduction of vasopressors and administration of an anticoagulant such as heparin (if HIT is not present). Other reported measures include sympathetic blockade, intravenously administered vasodilators, local injection or intravenous infusion of alpha-blockers, and intravenously administered prostaglandins, but with variable (usually minimal) degree of success. Other maneuvers to prevent DVT/VLG include intermittent calf compression, extremity elevation, fluid resuscitation, and avoidance of warfarin. Thrombolysis with urokinase is another potential therapy. If thrombolysis is contraindicated (as in our patient case), surgical venous thrombectomy plus or minus distal arteriovenous fistula is an option to promote recanalization [19]; if compartment syndrome develops, fasciotomy is indicated.

### Conclusion

The occurrence of DVT in a patient with post-trauma SPG was associated with greater ischemic limb injury in the limb affected by the DVT. Thus, our patient appears to have developed SPG (critical illness, hypotension resulting in shock liver, hypercoagulability state with dropping platelet count, and bilateral lower limb ischemic injury despite presence of arterial pulses) together with VLG (that is, presence of DVT in a limb with ischemic necrosis despite presence of arterial pulses). He developed a greater degree of ischemic injury in the limb affected by DVT, indicating that concomitant DVT can modulate the clinical course of SPG.

## Abbreviations

BP: Blood pressure
CRP: C-reactive protein
CT: Computed tomography
DIC: Disseminated intravascular coagulation
DVT: Deep vein thrombosis
GCS: Glasgow Coma Scale
HIT: Heparin-induced thrombocytopenia
INR: International normalized ratio
IV: Intravenous
SPG: Symmetrical peripheral gangrene
VLG: Venous limb gangrene
